# Development of the Digital Inclusion Questionnaire (DIQUEST) in Parkinson’s Disease

**DOI:** 10.1007/s10072-023-07090-3

**Published:** 2023-10-16

**Authors:** Vincenzo Canoro, Marina Picillo, Sofia Cuoco, Maria Teresa Pellecchia, Paolo Barone, Roberto Erro

**Affiliations:** Department of Medicine, Surgery and Dentistry “Scuola Medica Salernitana”, Neuroscience Section, 84081 Baronissi, SA Italy

**Keywords:** Telemedicine, Telehealth, Digital technologies, ICT, Remote visits

## Abstract

**Background:**

No tool is currently able to measure digital inclusion in clinical populations suitable for telemedicine. We developed the “Digital Inclusion Questionnaire” (DIQUEST) to estimate *access* and *skills* in Parkinson’s Disease (PD) patients and verified its properties with a pilot study.

**Methods:**

Thirty PD patients completed the initial version of the DIQUEST along with the Mobile Device Proficiency Questionnaire (MDPQ) and a practical computer task. A Principal Components Analysis (PCA) was conducted to define the DIQUEST factor structure and remove less informative items. We used Cronbach’s *α* to measure internal reliability and Spearman’s correlation test to determine the convergent and predictive validity with the MDPQ and the practical task, respectively.

**Results:**

The final version of the DIQUEST consisted of 20 items clustering in five components: “advanced skills,” “navigation skills,” “basic skills/knowledge,” “physical access,” and “economical access.” All components showed high reliability (*α* > 0.75) as did the entire questionnaire (*α* = 0.94). Correlation analysis demonstrated high convergent (rho: 0.911; *p*<0.001) and predictive (rho: 0.807; *p*<0.001) validity.

**Conclusions:**

We have here presented the development of the DIQUEST as a screening tool to assess the level of digital inclusion, particularly addressing the *access* and *skills* domains. Future studies are needed for its validation beyond PD.

**Supplementary Information:**

The online version contains supplementary material available at 10.1007/s10072-023-07090-3.

## Introduction

Telemedicine or “healing at a distance” is a broad concept encompassing all kinds of medical acts which are performed remotely [1]; it implies connecting patients with healthcare professionals using digital information and communication technologies (ICTs) that, nowadays, make not only use of computers and internet networks, but also mobile devices like tablets, smartphones, and wearables [1, 2]. Telemedicine can include different types of services, either with synchronus or asynchronus delivery (e.g., videconference-based visits vs mail exchanges) that can be variably used according to the specific needs of different clinical populations [3].

Following the COVID-19 pandemic, there has been a significant boost of telemedicine services, with the percentage of US older adults receiving a remote visit raising from 4% in May 2019 to 30% in June 2020 [4]. Indeed, some have argued that the pandemic should represent a “catalyst” for increasing the use of telemedicine in neurological care [5]. Such conditions as Parkinson’s Disease (PD) are well suited to telemedicine because they are primarily visually assessed, limit mobility, and require ongoing multidisciplinary care [6–10].

Even before the pandemic, studies have demonstrated the feasibility of using telemedicine services and highlighted many advantages from both PD patients’ and physicians’ perspective [10–13]. Neurologists can administer most of the “Movement Disorders Society - Unified Parkinson’s Disease Rating Scale - part three” (MDS-UPDRS-III) by visual examination alone, except for tone and postural instability [7, 14, 15]. Moreover, it has been argued that observing patients in their environment may more accurately reflect their actual state than in the clinic as they tend to outperform during hospital consultations [10]. Patients’ satisfaction also seems high, main reasons being mostly related to time- and cost-savings [11–13, 16–18].

However, the increasing use of telemedicine services has also led to the identification of many barriers including costs, reimbursement, confidentiality concerns, and negative perceptions [6, 8, 10]. Moreover, especially for adults >65 years, there were known to be additional challenges such as lower technological literacy [19, 20]. Indeed, in order to efficiently use telehealth services, patients need to be proficient with digital technologies and this prerequisite is not always satisfied. Despite worldwide spreading of digital devices, there exists a gap between subjects who are able to exploit them and those who are not, a phenomenon that has been termed “digital exclusion” [21–23].

The main factors contributing to the level of digital inclusion are access to technologies, skills in using ICTs, and attitude to use ICTs [22]. Typically, disadvantaged groups include less educated, disabled, elderly people, and those with low socio-economic status [20–23].

While previous studies in PD have shown the feasibility of delivering telemedicine, further demonstrating positive outcomes in patients using such services [6], none has investigated the level of digital inclusion of this population and this is an essential step for wide implementation of remote services in clinical practice. Although several scales have previously been proposed to assess digital abilities [19, 24–28], to the best of our knowledge none has been explicitly developed for telemedicine as they were originally conceived for other purposes (e.g., screening tools for digital training classes) [26, 27]. Moreover, these tools are mainly focused on digital literacy, which represents only one of the barriers contributing to the digital exclusion [23]. Therefore, in order to explore this issue in a more comprehensive way in the context of a clinical population such as PD, we developed a new tool named “Digital Inclusion Questionnaire” (DIQUEST). Namely, we were interested in capturing potential barriers to telemedicine use relating to both access and skills. At this stage, while developing the questionnaire, we did not include items measuring “attitude” because of the fact that some intrinsic features of the disease such as mood disturbances and apathy could significantly confound the results [29, 30]. Moreover, in general terms, many studies have already demonstrated a good propensity of elderly towards telemedicine [14, 31, 32].

Therefore, in this study, we present the development of the DIQUEST and its properties (factor structure, reliability, convergent validity, and predictive validity) after administration in a sample of consecutive PD patients, a peculiar clinical population that might grossly benefit from telehealth.

## Materials and methods

The investigators developed an initial version of the DIQUEST with 30 items, some of which were adapted from the Internet Skills Scale (ISS), a validated measure aimed at estimating internet skills in nationally representative surveys [28]. From the full ISS, we removed two sections (namely “social” and “creative”) that we considered to be outside the scope of this study as they explore capabilities that do not directly impact the use of telehealth services. Additional items were then developed to explore other potential barriers to the use of telehealth: namely, access to computers, internet connection, and smartphones; basic knowledge; frequency of use and specific abilities in employing video-calling applications from both computers and smartphones, since they require different methods and dedicated softwares (see Supplementary Table 1)*.* For each item, subjects had 2 to 6 possible answers, each with different scores assigned: higher scores were associated with better capabilities, hence reflecting higher digital inclusion levels.

Consecutive PD patients without overt cognitive and mood disturbances, attending our outpatient clinic for regular follow-up visits over a 2-month period, were recruited and were asked to complete the initial version of the DIQUEST as well as to fill in the Mobile Device Proficiency Questionnaire (MDPQ) [27] and to perform a practical computer task, as described below.

Both questionnaires were administered as paper version by the same expert examiner with clear explanation of each item: this ensured consistency in scoring. Additionally, demographic (age, sex, housing context, and employment) and clinical information (MDS-UPDRS-III [33] and Hoehn and Yahr staging [34]) were concomitantly gathered.

After collecting the data using the initial version of the DIQUEST, a Principal Components Analysis (PCA) using Varimax Rotation was conducted in IBM SPSS Statistics (Version 25) to define the factor structure of our questionnaire and remove less informative and redundant items, adopting an a priori cut-off value of rotated components’ matrix coefficients > 0.70. This allowed to obtain the final version of the DIQUEST.

The convergent validity of the DIQUEST with other scales assessing digital skills was then measured with the MDPQ. We adopted this scale over others because of the increasing use of mobile devices such as smartphones for delivering telemedicine services.

Finally, as mentioned above, the subjects were asked to perform a practical computer task. This was developed to measure the predictive validity of the DIQUEST. Namely, we asked subjects to complete a multi-step process with the ultimate aim of downloading a password-secured clinical letter of a putative teleconsultation that was sent via e-mail. Subject had to use a Windows PC to access an e-mail box created specifically for this study. Login credentials (a username and a password with upper and lower case letters and special characters) were exhibited on a printed paper. Once logged, subjects could find two messages in the mailbox: one with attached the password-secured clinical letter (a .pdf document) and in which they were instructed that the password would have been received with a second email and a second one carrying in attachment a text file (.doc) containing the password. Subjects had to copy and paste or write mnemonically the password to open the PDF file. The latter contained the actual instruction to end the task, that is to delete both files and to log out of the e-mail box. Each of this operation was scored from 0 to 1—with the sole exception of two items (*download the second attachment* and *logout*) whose maximum score was 0.5—obtaining a final score with a maximum of 10. No time limit was imposed; however, when patients indicated that they could not proceed in any of the aforementioned steps, the operation was performed by the examiner and the test continued, assigning a score of 0 for that specific step (see Supplementary Table 2).

Qualitative variables such as employment and housing context were converted into ordinal ones, with higher values indicating job status with higher income (i.e., 1= unemployed, 2= retired, 3= part-time employee, 4= full-time employee, 5= self-employed) and urban housing (i.e., 1= nursing homes, 2= rural context, 3= semi-rural context, and 4= urban context), respectively. A new variable called “employment x housing” was created by multiplying the two aforementioned ordinal variables with the intention to capture the overall individual economic status.

Statistical analyses have been made using IBM SPSS Statistics (Version 25). Mean and standard deviation are displayed for variables with normal distribution, median and interquartile range (IQR) for non-parametric variables; Cronbach’s *α* was used to assess reliability; Spearman’s correlation tests were conducted to determine relations between variables of interest.

## Results

### Sample population

Thirty PD patients (27 male and 3 female) with a mean age of 65.1 ± 7.7, mean education of 12 ± 4 years, and a mean MDS-UPDRS-III of 23.1 ± 8.7 were enrolled. The median H&Y was 2 (IQR = 0.5). As expected, most of our subjects were retired (19 out of 30, 66.7%) with 1 subject being unemployed (3.3%), 6 full-time employed (20%), and 4 self-employed (13.3%). Sixteen patients (53.3%) lived in an urban context, whereas 2 (6.7%) in a rural and 12 (40%) in a semi-rural context. The median score for the computer task was 1.75 (IQR = 6.1); the median MDPQ score was 114 (IQR = 87.25).

### Principal Components Analysis and definition of the final structure of the questionnaire

There were no missing data in the initial DIQUEST and after conducting the PCA, twenty items were retained from the initial pool, according to the a priori selected coefficient above 0.70 in the rotated components matrix. Accordingly, the final version of the DIQUEST is constituted of 5 main components (3 skills subscales and 2 access subscales; see Table 1), as follows: component 1 included five items referring to more *advanced skills* (i.e., using shortcut keys, bookmarking a website, downloading and installing smartphone apps, using video-calling softwares on a computer) than items in component 2 that are focused on *navigation skills* only, as well as those in component 3 which included four items reflecting *basic skills/knowledge*; the last two components included questions relative to *physical access* (component 4, three items) and *economical access* (component 5, two items) to a computer and to an internet connection. The final version of the DIQUEST was used for subsequent analyses, as continued below.

**Table 1:** Final version of the DIQUEST; coefficients in bold highlight items clustering as resulted from Principal Components Analysis (PCA)

Items	Components				
	1	2	3	4	5
Could you afford a 500€ computer?	0.101	0.067	−0.014	0.07	**0.953**
Could you afford an Internet connection with an average cost of 30€ per month?	0.09	0.056	0.022	0.261	**0.901**
I have a computer at home	0.168	0.125	0.083	**0.803**	0.308
I have a computer that is not shared with others	0.263	0.18	0.117	**0.737**	−0.139
I have a (good) internet connection	0.053	0.231	0.167	**0.802**	0.329
Have you ever used a computer?	0.311	0.177	**0.825**	0.313	0.069
How long have you been using a computer?	0.412	0.189	**0.777**	0.337	0.031
Are you able to turn on a computer?	0.401	0.202	**0.786**	0.315	0.123
Do you know what video calling applications are?	0.321	0.157	**0.755**	−0.087	−0.166
Are you able to launch a video calling application from a computer?	0.244	**0.779**	0.093	0.401	0.028
I know how to use shortcut keys (e.g., CTRL-C for copy, CTRL-S for save)	0.289	**0.803**	0.131	0.233	−0.111
I know how to bookmark a website	0.37	**0.765**	0.269	0.299	0.167
I know how to install apps on a mobile device	0.595	**0.724**	0.166	0.015	0.07
I know how to download apps to my mobile device	0.577	**0.727**	0.216	−0.029	0.098
How many times do you surf the internet in a week?	**0.827**	0.226	0.27	0.193	0.158
Are you able to access the internet from a smartphone?	**0.833**	0.353	0.339	0.091	0.003
I find it hard to decide what the best keywords are to use for online searches	**0.793**	0.3	0.38	0.293	0.01
I find it hard to find a website I visited before	**0.718**	0.221	0.238	0.458	0.106
I get tired when looking for information online	**0.866**	0.221	0.313	0.094	−0.015
Sometimes I end up on websites without knowing how I got there	**0.706**	0.412	0.384	0.305	0.11

### Reliability

Reliability analysis revealed strong internal consistency: Cronbach’s alpha was 0.94 for the entire questionnaire and above 0.75 for each of the five components (i.e., advanced skills *α* = 0.80; navigation skills *α* = 0.97; basic skills/knowledge *α* = 0.83; physical access *α* = 0.78; economical access *α* = 0.80).

### Correlation analysis and validity

The DIQUEST score showed significant correlation with education (rho=0.576; *p*=0.001), age (rho=−.399; *p*=0.029), and employment x housing (rho=0.524; *p*=0.003), whereas it did not correlate with motor disability as assessed by the MDS-UPDRS-III (rho=−.227; *p*=0.228)

**Fig. 1:**
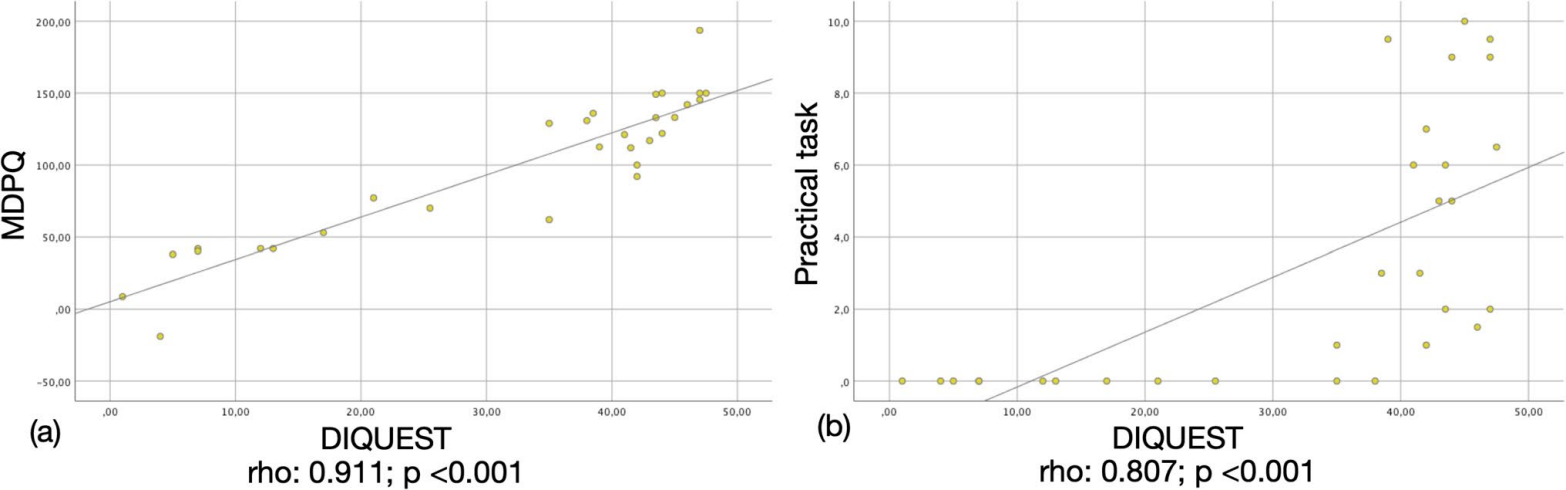
Scatterplots showing the relationship between DIQUEST total score and either (**a**) MDPQ or (**b**) practical task

To assess convergent validity, further correlation analyses were conducted between the MDPQ score and either total or individual domains’ score of the DIQUEST. A strong convergent validity was found between the MDPQ and the total DIQUEST score (rho=0.911, *p* <0.001; Fig. 1a) as well as with all its components (advanced skills: rho=0.886; *p* <0.001; basic skills/knowledge: rho=0.684; *p* <0.001; navigation skills: rho=0.851; *p*<0.001; physical access: rho=0.546; *p*=0.002; see also Supplementary Fig. 1) but the component assessing economical access (rho=0.303; *p*= 0.103).

The predictive validity of the DIQUEST was found to be good since it strongly correlated with the performance at the practical task (rho=0.807; *p*<0.001; Fig. 1b) as all its components did (advanced skills: rho=0.806; *p*<0.001; basic skills/knowledge: rho=0.691; *p* <0.001; navigation skills: rho=0.717; *p* <0.001; physical access: rho=0.632, *p*<0.001; see also Supplementary Fig. 1) but the one assessing the economical access (rho=0.104; *p*=0.583). The validity of the practical task was confirmed by its strong correlation with the MDPQ score (0.717; *p*<0.001).

## Discussion

For wide implementation of telemedicine services, it is fundamental to estimate patients’ level of digital inclusion to overcome potential barriers; unfortunately, no dedicated tool has been yet conceived for its quantification [35]. In fact, the only scale specifically developed for telemedicine is the “Telehealth Readiness Assessment Tool,” the purpose of which is to assess the ability of health institutions to initiate remote services and it is intended to be filled in by healthcare professionals [36]. Although it includes a “patient section” with reference to the average catchment area, it does not specifically measure the level of digital inclusion either at group or individual level [36]. Other existing scales mainly focus on digital literacy, therefore assessing only one potential barrier to the use of telemedicine [19, 24–28]. When developing the DIQUEST, we intended to create a tool short enough to be easily administered for screening during a routine visit and able to assess the two main domains contributing to digital exclusion in clinical populations (i.e., access and skills).

The DIQUEST was shown to highly correlate with the MDPQ [27], demonstrating a strong convergent validity. Since the MDPQ only measures digital skills, we expected to see highest coefficients with the items belonging to the first three components (i.e., advanced skills, navigation skills, and basic skills/knowledge) and the results supported our expectations. The total DIQUEST score also strongly correlated with the variable assessing individual economic status, implying it correctly identifies potential barriers related to limited access to ICTs. This is in line with previously published research [37–39], which further shows how other demographic factors such as age and education contribute to the level of digital inclusion as it happened in our case. However, in this study, we used a single variable to combine two different information (employment status and housing context), which does not allow to to clearly understand how much these might weigh individually.

The DIQUEST also showed good predictive validity since it strongly correlated with the performance at a practical task. This is a strength of our study in comparison to previous research that has used self-reported experience as the reference [26, 27]. We adopted this approach in order to avoid overestimation of one’s digital skills that might derive from limited digital literacy. It should be noted however that there was some degree of disagreement between the two measures with some subjects showing a low performance at the practical computer task even with relatively high DIQUEST scores. This might be explained by the fact that (1) the DIQUEST assesses not only digital skills but also access to ICTs, (2) there might exist a difference in the proficiency in using different devices in the way that smartphones might be more easy to use and the DIQUEST also includes items assessing smartphone use, and (3) some subjects might be able to perform some computer tasks only when certain conditions are satisfied (for instance, connecting to the mailbox they are used to and/or with auto-saved credentials or with the assistance of a caregiver/family member).

Future research applying the DIQUEST in a large sample of PD patients might reveal whether divergent digital proficiency exists between desktop and mobile devices. If so, a further refinement of the DIQUEST might be envisioned to focus more specifically on either device, in consideration of the fact that the use of mobile devices is now more widespread than the use of computers, but also that widely used apps, including those for video-calls, do not ensure data security, privacy, etc. that are instead fundamental for telemedicine services.

We also acknowledge that the broader concept of digital inclusion also encompasses the attitude to use ICTs [23]. If on the one hand our DIQUEST does not capture this aspect, previous research has demonstrated a good propensity of PD patients towards telemedicine services [16–18], although these findings were obtained from samples mainly enrolled through online survey which might have created a recruitment bias. This aspect should be therefore deepened in subsequent researches. Finally, we acknowledge that our, relatively limited, sample cannot be deemed representative of the whole PD population and that there was an imbalance in terms of sex, a factor that has been shown to influence to some degree the level of digital inclusion [40].

The results of our pilot study suggest that the DIQUEST could be a short instrument able to capture the two main components of digital inclusion that most affect the use of telemedicine services. However, due to the aforementioned limitations, in order to ensure the generalizability of the results and obtain a complete validation of the tool, further studies are needed: these should involve larger PD populations, including also more advanced patients who may mostly benefit from remote consultations. Moreover, since the DIQUEST is a generic scale, dedicated testing could also be envisioned in clinical populations other than PD patients, such as those with related parkinsonian disorders, for which our tool could be equally suitable.

### Supplementary information

ESM 1 (DOCX 434 kb)

## Data Availability

The datasets generated and/or analyzed during the current study are available from the corresponding author on reasonable request.
