# Supplementary material for: Development of the Digital Inclusion Questionnaire (DIQUEST) in Parkinson’s Disease
Source: Neurol Sci. 2023 Oct 16;45(3):1063–9. doi: 10.1007/s10072-023-07090-3 (PMC10857963; doi:10.1007/s10072-023-07090-3)
Supplement: Supplementary file 1 — (DOCX 434 kb) [file 10072_2023_7090_MOESM1_ESM.docx]

**Development of the Digital Inclusion Questionnaire (DIQUEST) in Parkinson’s Disease**

Neurological Sciences

Vincenzo Canoro, MD, Marina Picillo, MD, PhD, Sofia Cuoco, PhD, Maria Teresa Pellecchia, MD, PhD, Paolo Barone, MD, PhD, Roberto Erro, MD, PhD

Correspondence to:

Roberto Erro

Department of Medicine, Surgery and Dentistry “Scuola Medica Salernitana”, Neuroscience section, University of Salerno

Via Allende, Baronissi, (SA) Italy

[erro.roberto@gmail.com](mailto:erro.roberto@gmail.com)

Tel.: +39-089673952

**Supplementary Table 1** Items included in the initial version of the DIQUEST.

| **FIELD** | **Items** |
| --- | --- |
| ACCESS | - I have a computer at home - I have a computer that is not shared with others - Could you afford a 500€ computer? - I have a (good) internet connection - Could you afford an Internet connection with an average cost of 30€ per month? - I have a smartphone - I have a contract on my smartphone with limited/unlimited internet access |
| SKILLS | - Have you ever used a computer? - How long have you been using a computer? - How many times do you surf the internet in a week? - How much time do you spend on the internet every time you surf? - Are you able to turn on a computer? - Are you able to access the internet from a computer? - Are you able to access the internet from a smartphone? - Do you know what video calling applications are? - Have you ever used video calling applications? - Are you able to launch a video calling application from a computer? - Are you able to launch a video calling application from a smartphone? |
| ISS-DERIVED ITEMS | OPERATIONAL   - I know how to open downloaded files - I know how to download/save a photo I found online - I know how to use shortcut keys (e.g. CTRL-C for copy, CTRL-S for save) - I know how to open a new tab in my browser - I know how to bookmark a website   INFORMATION NAVIGATION   - I find it hard to decide what the best keywords are to use for online searches - I find it hard to find a website I visited before - I get tired when looking for information online - sometimes I end up on websites without knowing how I got there   MOBILE   - I know how to install apps on a mobile device - I know how to download apps to my mobile device - I know how to keep track of the costs of mobile app use |

**Supplementary Table 2** Items of the practical computer task and relative scoring system.

| **Item** | **Score range** |
| --- | --- |
| Recognizes and launches the browser from the home screen | Yes - 1  No - 0 |
| Reaches the mailbox provider via the search/address bar | Yes - 1  No - 0 |
| Inserts access credentials (alphanumeric password with special characters) | Yes - 1  No - 0 |
| Opens the message and downloads the attachment (1 of 2) | Yes - 1  No - 0 |
| Opens the message and downloads the attachment (2 of 2) | Yes - 0.5  No - 0 |
| Opens the *Download* folder | Yes - 1  No - 0 |
| Copies the password from the .txt file | Yes - 1  No - 0 |
| Pastes the password where requested to open the .pdf file | Yes - 1  Writes from memory - 0.5  No - 0 |
| Opens the .pdf file | Yes - 1  No - 0 |
| Deletes downloaded files | Yes - 1  No - 0 |
| Logs out from the mailbox | Yes - 0.5  No - 0 |

**Supplementary** **Fig.1** Scatterplots showing relationship between DIQUEST’s components’ scores and either (**a-d**) MDPQ or (**e-h**) practical task
